# Triple-Vessel Disease in a High-Risk Surgical Patient Treated With Nine Drug-Eluting Stents

**DOI:** 10.7759/cureus.60791

**Published:** 2024-05-21

**Authors:** Usman S Najam, Zain Ali

**Affiliations:** 1 Internal Medicine, University at Buffalo Jacobs School of Medicine and Biomedical Sciences, Buffalo, USA; 2 General Medicine, Khyber Medical College, Peshawar, PAK

**Keywords:** drug-eluting stents, high-risk percutaneous coronary intervention, adult cardiology, multivessel coronary artery disease (mvcad), interventional cardiology

## Abstract

Coronary artery disease (CAD) is a major cause of morbidity and mortality in the United States, and as strides have been made in its management, outcomes have continued to improve. Management has evolved from expectant management to coronary artery bypass graft surgery and thrombolysis, to more recently percutaneous intervention with stenting and medical management in select cases. Here, we describe a case of a complex patient with severe triple-vessel disease who was deemed a poor surgical candidate for coronary artery bypass graft surgery and would instead undergo high-risk percutaneous intervention with the placement of nine drug-eluting stents.

## Introduction

Coronary artery disease (CAD) is the leading cause of mortality in the United States and the third leading cause worldwide [1]. Its management costs the healthcare industry billions of dollars per year and causes significant emotional distress to those involved [1]. It is a multifactorial disease caused by the buildup of cholesterol in the coronary arteries, leading to progressive flow-limiting stenosis over time [2]. After a diagnosis of CAD is made, pursuing the appropriate management is paramount, as improper management may lead to arrhythmias, heart failure, and other life-threatening sequela. Treatment has constantly been evolving, from expectant management to coronary artery bypass graft surgery (CABG) and thrombolysis, to most recently percutaneous intervention (PCI). In patients without significant disease, medical management may be the indicated treatment, while in others with more severe disease, PCI or CABG has proven to improve outcomes [2]. Current evidence supports the use of CABG in patients with left main, severe three-vessel, and diffuse diseases, among other reasons, while PCI is used in the majority of cases who do not meet the requirement for CABG. In complex patients who are found to have severe coronary disease, but whose risk factors preclude surgery, a specialized high-risk PCI can be performed. High-risk PCI, per the Interventional Council of the American College of Cardiology, is a PCI that is performed on patients who have an unprotected left main disease, left ventricular ejection fraction <35%, three-vessel CAD, severe aortic stenosis, severe mitral regurgitation, or a last remaining conduit [3]. High-risk PCI has continued to gain support over previous years, with our case being a prime example of how a complex patient with severe triple-vessel disease can be managed with a high-risk PCI.

## Case presentation

**Figure 1:**
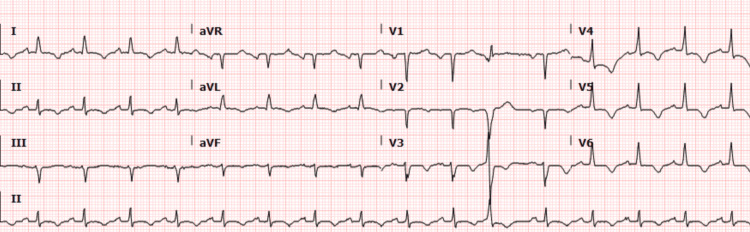
ECG showing sinus rhythm with occasional premature complexes and anterolateral T-wave inversions

A 76-year-old male, with a past medical history of type II diabetes mellitus, hyperlipidemia, and hypertension, presented with complaints of chest pain and dyspnea for the past two weeks. The patient denied any previous history of acute coronary syndrome, cardiac interventions, heart failure, or previous blood clots. He denied any recent travel or sick contacts, as well as denied anything that exacerbated or improved his symptoms. He stated he had been having dyspnea with increased difficulty ambulating for about six months, often having to stop to catch his breath. However, more recently, he began having dull chest pain, which started about two weeks ago and had been intermittent. The patient initially believed his symptoms were musculoskeletal; however, after continued symptoms, he was urged by his son to visit the hospital. The patient's medication regimen was most notable for an insulin regimen for his diabetes and amlodipine for hypertension. His initial assessment was primarily concerning for acute coronary syndrome, with differentials including a pulmonary embolism, aortic dissection, vasospastic angina, heart failure exacerbation, hypertensive emergency, etc. His physical exam was largely unremarkable. However, fine expiratory crackles were noted at bilateral lung bases. An EKG demonstrated T-wave inversions in the anterolateral leads (Figure 1). However, no ST segment changes, and his chest X-ray showed mild bilateral pleural effusions and diffuse pulmonary edema. A stat echocardiogram was performed and showed a left ventricular ejection fraction of 20-25% (Video 1). The patient's labs returned at this point, which was most notable for a troponin elevation of 0.33 and brain natriuretic peptide >9,000. The on-call interventional cardiologist was called and agreed to take the patient to the cardiac catheterization lab.

**Video 1:** Echocardiogram with apical four-chamber view showing an ejection fraction (EF) of 20-25%

**Figure 2:**
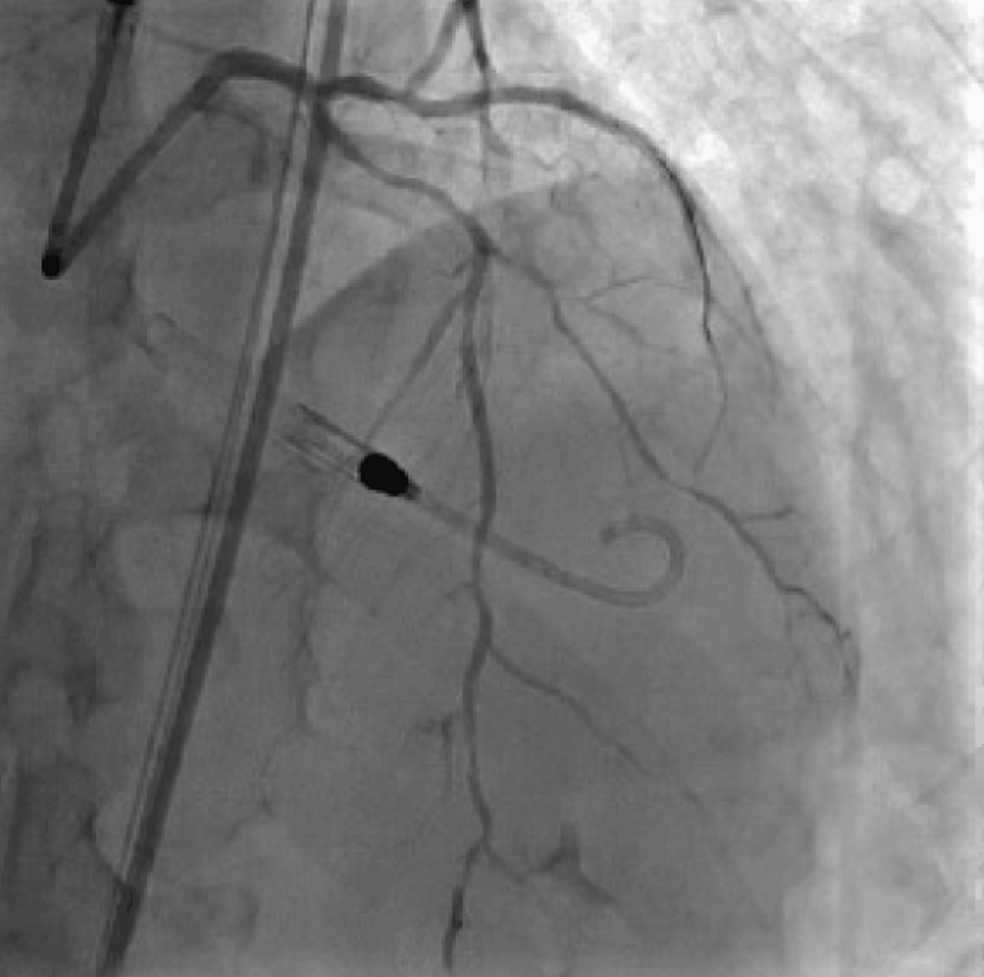
Diseased left anterior descending artery and first diagonal artery with flow-limiting lesions

**Figure 3:**
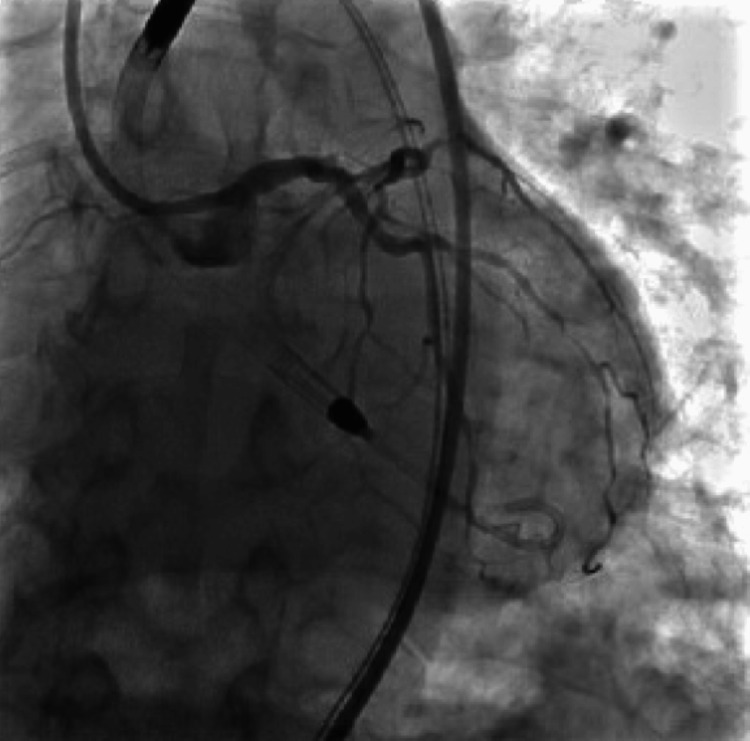
Diseased obtuse marginal artery with flow-limiting lesions

**Figure 4:**
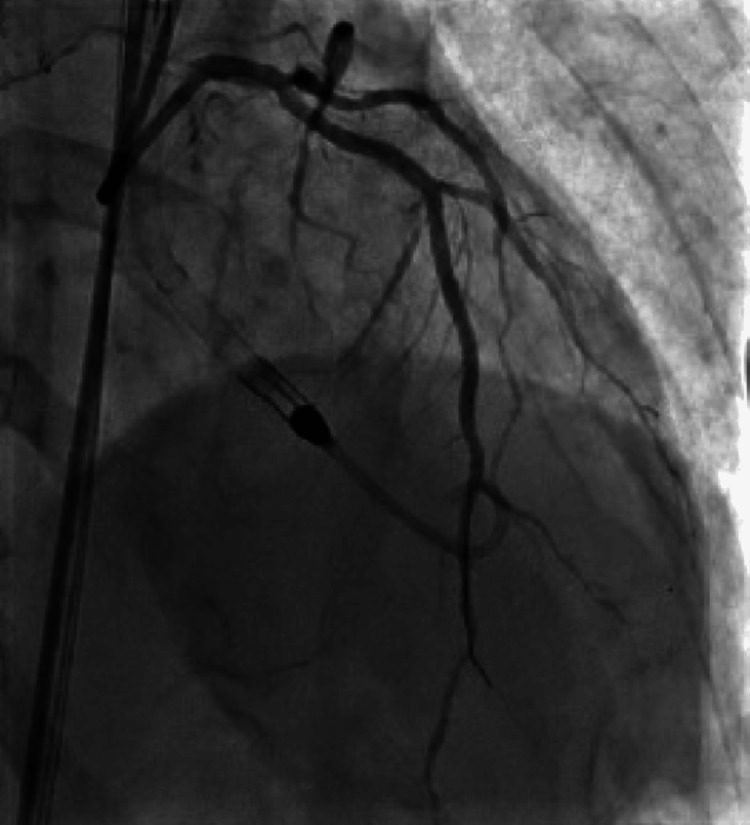
Left anterior descending artery and first diagonal artery after percutaneous intervention with one drug-eluting stent in the proximal first diagonal branch and four drug-eluting stents to the proximal to the distal-apical left anterior descending artery

**Figure 5:**
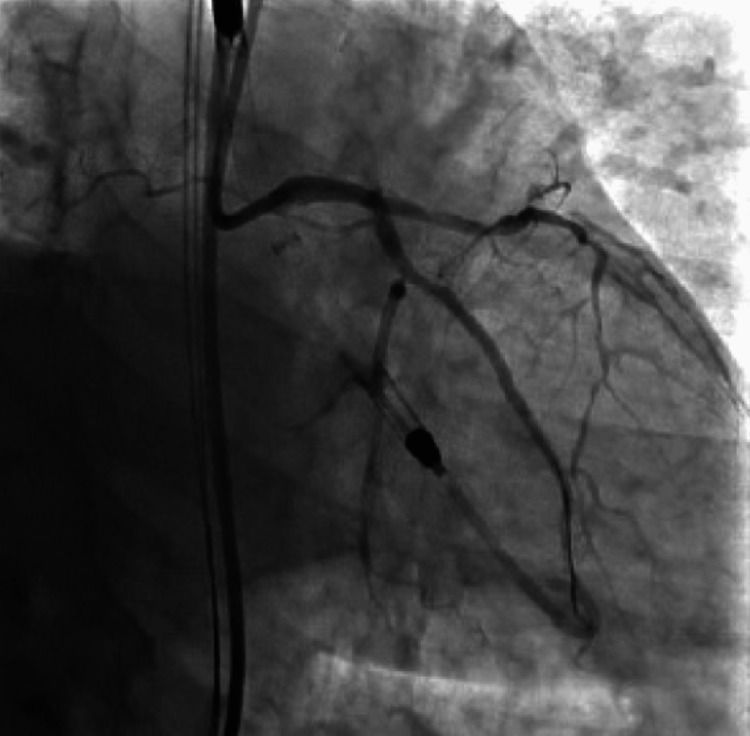
First obtuse marginal artery with two drug-eluting stents placed

**Figure 6:**
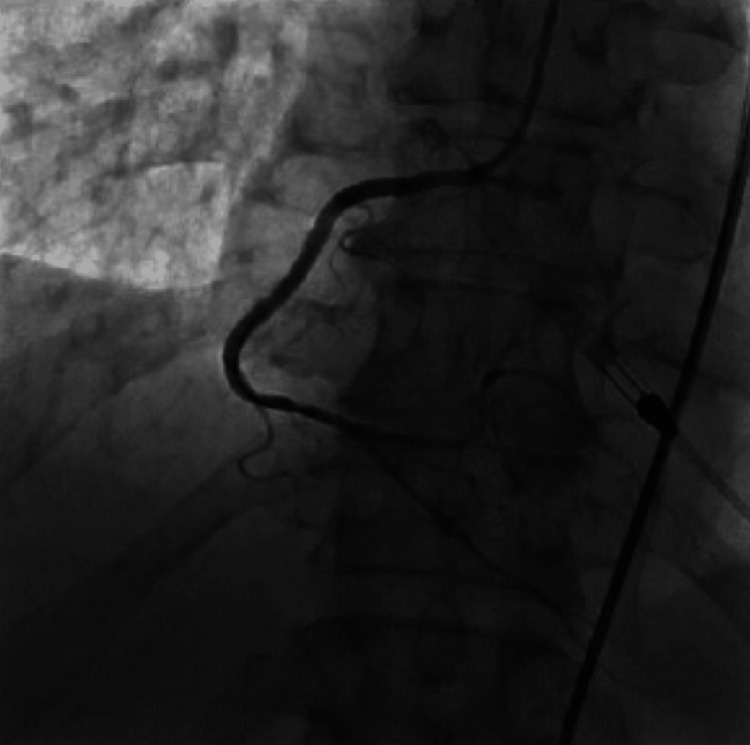
Right coronary artery after two drug-eluting stents placed

During the procedure, the patient was found to have severe triple vessel disease involving his right coronary artery (RCA), proximal to distal-apical left anterior descending (LAD) artery, and its first diagonal branch (D1) (Figure 2), as well as the first obtuse marginal branch (OM1) of the left circumflex artery (LCx) (Figure 3). The patient's severe triple-vessel disease warranted evaluation for CABG, and no intervention was done at that time. The patient was evaluated by the cardiothoracic surgery team, who calculated the patient's predicted risk of mortality, which was 15.7% by the European System for Cardiac Operative Risk Evaluation (EuroSCORE) II and 14.1% using the Society of Thoracic Surgeons (STS) operative risk assessment. A multidisciplinary meeting was held, with the CT surgery and interventional cardiology team deciding due to the patient's coronary artery anatomy, comorbidities, and acuity, that he would be a poor surgical candidate for CABG. The patient was explained his options, whether it be medical management or high-risk PCI, along with the potential risks and benefits of both. He was explained that, although performing PCI with stent placement could potentially help with symptoms and quality of life, it would nonetheless carry a substantial amount of risk. He would have the risk of intra-procedural complications, such as coronary artery rupture, dissections, infection, bleeding at the catheter site, etc. Additionally, after multiple stents are placed, the patient would need to remain on dual anti-platelet therapy for an extended period of time with a higher-than-usual risk of in-stent restenosis, particularly with full metal jacket stenting in which multiple stents would be placed in succession due to his extensive lesions. After explaining the additional risks and benefits, the patient expressed understanding and agreed to undergo the procedure. The patient was taken for the procedure a week later, and under Impella support, received a total of nine drug-eluting stents, four to the proximal to the distal-apical LAD artery and one to the D1 branch (Figure 4), two to the OM1 branch (Figure 5), and two to the RCA (Figure 6). At the end of the procedure, the Impella was removed, and the patient was taken to the cardiac critical care unit.

After arriving at the unit, the patient felt well. However, he did become slightly hypotensive, requiring norepinephrine for hemodynamic support. However, over the next 24 hours, the patient's blood pressure improved, and his norepinephrine was weaned. His goal-directed medical therapy was optimized, which included starting the patient on high-dose atorvastatin, lisinopril, and metoprolol succinate. He was placed on ticagrelor and aspirin for his dual antiplatelet therapy. After being monitored for 48 hours in the cardiac critical care unit, the patient was transferred to the general cardiology service, where he remained for an additional four days to optimize his medical therapy and obtain a follow-up echocardiogram, which showed an ejection fraction of 25%, similar to that on arrival. The patient was ultimately discharged to a subacute rehab facility on dual antiplatelet therapy, sacubitril/valsartan, and metoprolol succinate. He was instructed to follow up with a cardiologist outpatient to further optimize his medications and monitor future symptoms.

## Discussion

CAD is a major cause of morbidity and mortality in the United States with its management often a topic of discussion. In patients without significant disease, medical management is often the indicated treatment. Meanwhile, PCI, when indicated, is focused on treating flow-limiting lesions found on coronary angiograms. However, when patients have severe coronary disease with the infarct being generated by non-flow limiting stenosis, CABG may be preferred [2]. In more complex cases, the management is often decided by a multidisciplinary team, with an unfortunate circumstance occurring in patients whose coronary disease would indicate CABG; however, their co-morbidities make them poor surgical candidates. In these patients, the prognosis is often very grim. However, with the ongoing progress in the prevention and treatment of CAD, significant advancements in treatment have been made, such as high-risk PCIs. Our patient exemplified this as he was not a candidate for CABG and underwent successful high-risk PCI. To appreciate how far the management of CAD has come, and to understand its future trajectory, it is important to see how management has changed over the past century.

Due to limitations in medical technology, before the 1960s, the management of CAD consisted of simple bed rest and a sodium-restricted diet [4]. A huge breakthrough occurred in 1961 when the first CABG was performed and published by Robert Goetz. Since then, CABG has continued to be one of the most common cardiac operations with several pioneers in the field contributing to its development [5]. The benefits of CABG stem from bypassing the entire diseased artery, which offers complete revascularization, providing a lower risk of heart attacks, and allowing patients to remain symptom-free for many years after a successful procedure [6]. The procedure is preferred in patients with triple vessel disease (two-vessel disease and diabetes), high-grade left main stem disease, severe left ventricular systolic dysfunction, etc.

In 1977 Dr. Andreas Gruentzig, a Swiss cardiologist, performed the first successful balloon angioplasty on a human patient. Over the years, advancements in catheter and guidewire technology have contributed to the refinements of PCI procedures [7]. The benefits of PCI include it being minimally invasive, relatively quick, and easy to perform, leading to a shorter hospital stay, and allowing patients to return to their daily life sooner after the procedure [8]. Initially, invasive procedures were performed on lower-risk patients, but with the sudden expansion of PCI throughout the years, high-risk PCI has been performed in select cases. High-risk PCI features are related to three clinical areas: patient risk factors and comorbidities, the complex coronary anatomy and location of the disease, and patient hemodynamic status. Along with the benefits of high-risk PCI, it is important to mention the pitfalls. Full metal jacket stenting, which is when extensive coronary lesions require multiple continuous overlapping stents, does not have sufficient data to show its safety or efficacy, and many who receive it have had poor short and long-term results [9]. These patients can often suffer from re-occlusion and in-stent thrombosis [9]. Treating high-risk patients is becoming more common and relevant in the modern day. However, as was done with our patient, a risk-benefit analysis with a multidisciplinary team is paramount for deciding its appropriateness [10].

An alternative management of CAD is medical therapy. Recently, the prognosis of stable CAD has improved due to advances in medical therapy. Medical therapy for CAD often includes lifestyle modifications, such as a heart-healthy diet, regular exercise, smoking cessation, and weight management, as well as various medications, including antiplatelet agents, statins to lower cholesterol, beta-blockers, angiotensin-converting enzyme (ACE) inhibitors, and antianginal medications. Patients may prefer these over procedures if there is a favorable prognosis as it lowers the cost of treatment and decreases the risk of adverse effects related to procedures [11].

The future management of CAD is very exciting and promising. The collaboration between technology and tissue engineering has led to the development of stem cells, nanotechnology, and robotics. The rationale behind stem cell therapy is to improve the blood supply to ischemic areas of the heart by stem cells, as well as promote cardiac cell regeneration [12]. Similarly, nanotechnology has shown potential benefits as they have been studied for their ability to release drugs, as well as promote healing and reduce restenosis [12]. Alternatively, robotics are being used for catheter-based surgical procedures and provide the operator with advantages such as improved ergonomics, precision, and sometimes shortening of intraoperative time and even shortening patient hospital stay [12]. As was seen in our case, the management of CAD is quickly evolving, and while high-risk PCI is not a mainstay in current management, the future remains very promising for it, as well as future techniques that will be developed.

## Conclusions

The management of CAD is an exciting and quickly evolving landscape. Since the implementation of CABG into medical practice, many changes have been made, and management is often made by a multidisciplinary team. Medical management remains a viable option for many. However, those with unrelenting angina and risk for re-infarction may require intervention. An unfortunate, yet common scenario is seen when a CABG candidate is not deemed a good surgical candidate. Medical management in this case may not be optimal as it can lead to increased morbidity and mortality. This case showed an example of a patient who was not a surgical candidate and went on to receive a high-risk PCI with a total of nine stents placed. Although placing a metal jacket in the coronary vessel with multiple stents can lead to an array of complications as described, in this case, the patient and providers believed it would allow the best chance of improvement, and a gamble was taken. As more high-risk PCIs are attempted and outcomes are tracked, we will have a greater understanding of their benefits and risks.
